# A Small Increase in Serum Creatinine within 48 h of Hospital Admission Is an Independent Predictor of In-Hospital Adverse Outcomes in Patients with ST-Segment Elevation Myocardial Infarction Undergoing Primary Percutaneous Coronary Intervention: Findings from the Improving Care for Cardiovascular Disease in the China Project

**DOI:** 10.1155/2023/1374206

**Published:** 2023-03-28

**Authors:** Jiajia Zhu, Wenxian Liu, Jiang Li, Changsheng Ma, Dong Zhao

**Affiliations:** ^1^Cardiac Intensive Care Unit, Beijing Anzhen Hospital, Capital Medical University, Beijing, China; ^2^Department of Cardiology, Beijing Anzhen Hospital, Capital Medical University, Beijing, China; ^3^Department of Epidemiology, Beijing Anzhen Hospital, Capital Medical University, Beijing Institute of Heart, Lung and Blood Vessel Diseases, Beijing, China

## Abstract

**Background:**

Acute kidney injury (AKI) is a common complication of percutaneous coronary intervention (PCI) that has been associated with high morbidity and mortality in patients with STEMI. Acute tubular damage may be reflected by serum creatinine (Scr) values that do not meet the criteria for AKI.

**Methods:**

This analysis included 19,424 patients from the Improving Care for Cardiovascular Disease in China, Acute Coronary Syndrome Project (*n* = 5,221 (36.8%), patients with a small increase in Scr within 48 h of hospitalization; *n* = 14,203 patients with no increase in Scr). The primary outcome was the incidence of major adverse cardiovascular events (MACE). Secondary outcomes included the incidence of massive hemorrhage, in-hospital death, atrial fibrillation, heart failure, cardiogenic shock, cardiac arrest, and stroke. Logistic regression analysis was used to evaluate associations between a small increase in Scr within 48 h of hospitalization (>0.1 to <0.3 mg/dl) and MACE or massive hemorrhage during hospitalization.

**Results:**

Patients with a small increase in Scr within 48 h of hospitalization were significantly more likely to experience MACE (11.2% vs. 9.1%; *P* < 0.001) or massive hemorrhage (3.2% vs. 2.2%; *P* < 0.001) compared to patients with no increase in Scr, but there was no significant difference in in-hospital mortality (0.8% vs. 0.9%; *P*=0.301). Logistic regression analysis showed that a small increase in Scr within 48 h of hospital admission was a risk factor for MACE (OR, 1.168; 95% CI, 1.044–1.306; *P*=0.006) or massive hemorrhage (OR, 1.413; 95% CI, 1.164–1.715; *P* < 0.001). Other risk factors included age ˃65 years, history of heart failure, use of glycoprotein IIb/IIIa inhibitors, aspirin or ACEI/ARB, LVEF <40%, Killip class III-IV, and increased SBP and heart rate.

**Conclusion:**

A small increase in Scr during hospitalization in patients with STEMI undergoing primary PCI that does not meet the criteria for AKI is a risk factor for in-hospital adverse outcomes. This effect is maintained in patients with normal Scr at hospitalization. *Trial Registration*. Clinical trial registration: URL: https://www.clinicaltrials.gov. Unique identifier: NCT02306616.

## 1. Introduction

An acute ST-segment elevation myocardial infarction (STEMI) is a serious form of the acute coronary syndrome (ACS) that can lead to sudden death. Mortality in patients with STEMI is reduced by the use of immediate revascularization. Acute kidney injury (AKI) is a common complication of percutaneous coronary intervention (PCI) that has been associated with high morbidity and mortality in patients with STEMI [1–5]. The occurrence of AKI increases the risk of death after STEMI by 3 to 5 times [6–9]. The important causes of AKI after PCI include renal damage from contrast medium and atherosclerotic embolism, cardiac insufficiency, and perioperative factors, such as the release of proinflammatory cytokines during surgery, postoperative endothelial dysfunction, platelet activation, and coagulation dysfunction. The incidence of AKI in patients with STEMI is estimated at 5.2% to 59.0% and varies with the measures used to diagnose AKI.

AKI has been defined and staged according to serum creatinine (Scr) levels. However, in clinical practice, serum creatinine (Scr) values increase with a delay of 48–72 h, following a renal injury, and acute tubular damage may be reflected by Scr that does not meet the criteria for AKI [10–12]. There is an unmet clinical need to understand the impact of small changes in Scr that do not meet the criteria for AKI on outcomes of patients with STEMI undergoing PCI. Previous studies investigating the relationship of small changes in Scr to in-hospital outcomes in patients after cardiac surgery or with STEMI undergoing PCI were single-center studies that did not consider the influence of important factors, including the use of angiotensin-converting enzyme inhibitors/angiotensin receptor blockers (ACEI/ARB). These medications are strongly recommended for patients with STEMI but may worsen the glomerular filtration rate (GFR) due to their effects on systemic blood pressure and efferent arteriolar tone [10–13].

The objective of the present study was to describe the incidence of small increases in Scr and its association with in-hospital outcomes in a large population of patients with STEMI undergoing primary PCI.

## 2. Materials and Methods

### 2.1. Data Source

This study included a consecutive sample of patients from the Improving Care for Cardiovascular Disease in China, Acute Coronary Syndrome Project (CCC-ACS Project), a nationwide registry and quality improvement study established among 150 hospitals that focus on the quality of care for patients with ACS. The CCC-ACS Project was launched in 2014 as a collaborative initiative of the American Heart Association and Chinese Heart Association. The details of the design and methodology of the CCC-ACS Project have been published elsewhere [14].

The data were abstracted from patients' medical records to a web-based data collection platform (Oracle Clinical Remote Data Capture; Oracle, Redwood Shores, CA, USA) by qualified staff. Audits were performed by third-party clinical research associates every 3 months on a random sample, representing >5% of the reported cases from every participating center to ensure data quality and accuracy. The audit reports confirmed that the data in this study were appropriately reported with an acceptable level of missing data or error.

The ethics committee of Beijing Anzhen Hospital, Capital Medical University, granted institutional review board approval for this study. Patients' written informed consent was not required for this study in accordance with the national legislation and the institutional requirements.

### 2.2. Study Population

Patients in the CCC-ACS Project with a principal discharge diagnosis of STEMI who underwent primary PCI between November 2014 and June 2017 were eligible for this study. STEMI was diagnosed in accordance with published guidelines. Criteria included a history of chest pain, diagnostic electrocardiographic changes, and serial elevation of cardiac biomarkers [15]. Patients with chronic kidney disease (CKD) (defined as a history of CKD or an estimated glomerular filtration rate (eGFR) of ≤60 ml/min/1.73 m^2^) at baseline, with a diagnosis of AKI within 48 h of hospital admission, who did not undergo primary PCI, or had incomplete SCr data were excluded from the analysis. The primary outcome was the incidence of major adverse cardiovascular events (MACE). Secondary outcomes included the incidence of massive hemorrhage and other adverse outcomes, including in-hospital death, stent thrombosis, atrial fibrillation (AF), heart failure (HF), cardiogenic shock (CS), and stroke. MACE was defined as a combination of these events.

**Figure 1:**
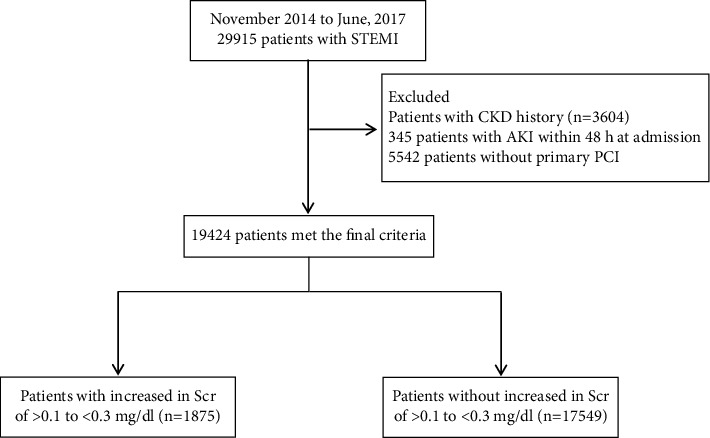
Flow chart.

Patients included in the final analysis were divided into two groups: those with or without a small increase in Scr within 48 h of hospital admission, defined as an increase in Scr of >0.1 to <0.3 mg/dl that did not meet the criteria for AKI [10] (Figure 1). AKI was defined as an increase in Scr of ≥0.3 mg/dl within 48 h of hospital admission according to the Kidney Disease Improving Global Outcomes (KDIGO) criteria [9].

### 2.3. Data Collection

Patients' medical records were reviewed and the following information was recorded: demographic characteristics; clinical characteristics, including cardiac enzymes, Killip class, blood pressure, comorbidities, medical history, findings on angiography, and invasive and noninvasive treatments; and in-hospital adverse events, including in-hospital death, stent thrombosis, AF, HF, CS, stroke, massive hemorrhage (defined as intracranial bleeding), retroperitoneal bleeding, a decline in hemoglobin of ≥4 g/dl during hospitalization, transfusion with overt bleeding, bleeding requiring surgical intervention [16], and the composite endpoint (MACE).

### 2.4. Statistical Analysis

The data were analyzed with SPSS v20.0 (IBM Corp., Armonk, NY, USA). Continuous variables are expressed as mean ± standard deviation and were compared with the Student's *t*-test or the Mann–Whitney *U* test, depending on the results of a test of normality. Categorical (frequency) variables are expressed as percentage and were compared using the Pearson's chi-square test. In the primary analysis, patients' demographic data, medical history, clinical treatment, and in-hospital outcomes were stratified by the presence or absence of a small increase in Scr within 48 h of hospital admission. Logistic regression analysis was used to evaluate associations between a small increase in Scr within 48 h of hospital admission and MACE or massive hemorrhage during hospitalization. Odds ratios (ORs) and 95% confidence intervals (CIs) were estimated. Multivariate logistic regression analysis included baseline variables with a *P* value <0.05 on univariate analysis. A subgroup analysis was performed in patients stratified by the presence of normal or abnormal Scr at baseline (hospital admission). Missing data were imputed with the sequential regression multiple imputation method using IVEware version 0.2 (Survey Research Center, University of Michigan, Ann Arbor, MI, USA). A two-tailed *P* value of <0.05 was considered statistically significant.

## 3. Results

### 3.1. Primary Analysis

A total of 29,915 patients in the CCC-ACS Project had a principal discharge diagnosis of STEMI between November 2014 and June 2017. Of these, 10,491 patients were excluded from this study, including 3,604 patients with chronic kidney disease (CKD) (defined as a history of CKD or an estimated glomerular filtration rate (eGFR) of ≤60 ml/min/1.73 m^2^) at baseline; 1,345 patients diagnosed with AKI within 48 h of hospital admission; and 5,542 patients who did not undergo primary PCI due to the risk of contrast-induced nephropathy. The final analysis included 19,424 patients with STEMI who underwent primary PCI, including 5,221 (36.8%) patients with a small increase in Scr within 48 h of hospital admission and 14,203 patients with no increase in Scr.

The baseline demographic and clinical characteristics of all patients with STEMI who underwent primary PCI are summarized in Supplementary Table 1. Patients with a small increase in Scr within 48 h of hospital admission were significantly older (61 vs. 60 y; *P* < 0.001), were significantly more likely to have hypertension (61.2% vs. 57.3%; *P* < 0.001) or dyslipidemia (86.1% vs. 81.1%; *P* < 0.001), had a significantly faster heart rate (79 vs. 77 bpm; *P* < 0.001), had a significantly higher creatine kinase-muscle/brain (CK-MB) and Scr and cardiac troponin I (TNI) levels, were significantly more likely to have a left ventricular ejection fraction (LVEF) <40% (9.5 vs. 7.0%), and were significantly more likely to be provided with glycoprotein IIb/IIIa inhibitors, ACEI/ARB, or beta blockers in-hospital compared to patients with no increase in Scr. Logistic regression analysis showed age >65 years, LVEF <40% and increased heart rate were significantly associated with a small increase in Scr within 48 h of hospital admission (Table 1).

In-hospital outcomes of all included patients are summarized in Table 2. Patients with a small increase in Scr within 48 h of hospital admission were significantly more likely to experience MACE (11.2% vs. 9.1%; *P* < 0.001), massive hemorrhage (3.2% vs. 2.2%; *P* <  0.001), or stent thrombosis (0.4% vs. 0.2%; *P*=0.004) compared to patients with no increase in Scr, but there was no significant difference in in-hospital mortality (0.8% vs. 0.9%; *P*=0.301).

Logistic regression analysis showed a small increase in Scr within 48 h of hospital admission was a risk factor for MACE (OR, 1.168; 95% CI, 1.044–1.306; *P*=0.006) or massive hemorrhage (OR, 1.413; 95% CI, 1.164–1.715; *P* < 0.001). Other risk factors included age ˃65 years, history of heart failure, use of glycoprotein IIb/IIIa inhibitors, aspirin or ACEI/ARB, LVEF <40%, Killip class III-IV, and increased SBP and heart rate (Tables 3 and 4).

### 3.2. Subgroup Analysis

#### 3.2.1. Patients with Normal Scr at Baseline

A total of 17,503 patients with STEMI who underwent primary PCI had normal Scr at baseline. Of these, 4,839 (27.6%) patients had a small increase in Scr within 48 h of hospital admission, and 12,664 patients had no increase in Scr (Supplementary Table 2). Patients with a small increase in Scr within 48 h of hospital admission were significantly older (61 vs. 60 y; *P* < 0.001), were significantly more likely to have hypertension (60.1% vs. 56.5%; *P* < 0.001) or dyslipidemia (86.2% vs. 82.4%; *P* < 0.001), had a significantly faster heart rate (79 vs. 77 bpm; *P* < 0.001), had significantly higher CK-MB and Scr and TNI levels, were significantly more likely to have LVEF <40% (9.2% vs. 6.4%; *P* < 0.001), and were significantly more likely to be provided glycoprotein IIb/IIIa inhibitors, ACEI/ARB, or beta blockers in-hospital compared to patients with no increase in Scr. Logistic regression analysis showed that age >65 years, use of glycoprotein IIb/IIIa inhibitors or anticoagulants, LVEF <40%, and increased heart rate were significantly associated with a small increase in Scr within 48 h of hospital admission (Supplementary Table 3).

In-hospital outcomes in patients with normal Scr at baseline are summarized in Supplementary Table 4. Patients with a small increase in Scr within 48 h of hospital admission were significantly more likely to experience MACE (10.6% vs. 7.9%; *P* < 0.001), heart failure (8.4% vs. 5.7%), or massive hemorrhage (2.9% vs. 1.9%; *P* < 0.001) compared to patients with no increase in Scr, but there was no significant difference in in-hospital mortality (0.7% vs. 0.6%; *P*=0.873). Logistic regression analysis showed a small increase in Scr within 48 h of hospital admission was an independent risk factor for MACE (OR, 1.247; 95% CI, 1.107–1.406; *P* < 0.001) and massive hemorrhage (OR, 1.445; 95% CI, 1.166–1.791; *P* < 0.001) (Supplementary Tables 5 and 6).

#### 3.2.2. Patients with Abnormal Scr at Baseline

A total of 1,921 patients with STEMI who underwent primary PCI had abnormal Scr at baseline. Of these, 382 (19.8%) patients had a small increase in Scr within 48 h of hospital admission, and 1, 539 (80.2%) patients had no increase in Scr (Supplementary Table 7). Patients with a small increase in Scr within 48 h of hospital admission were significantly older (66 vs. 65 y; *P* < 0.001), were significantly more likely to have hypertension (74.9% vs. 63.6%; *P* < 0.001) or dyslipidemia (84.0% vs. 76.7%; *P*=0.002), had a significantly faster heart rate (79 vs. 77 bpm; *P* < 0.001), had significantly higher Scr levels, and were significantly more likely be provided aspirin (*P*=0.025) or statins (*P*=0.002) in-hospital compared to patients with no increase in Scr. There were no significant differences in in-hospital outcomes in patients with a small increase in Scr within 48 h of hospital admission compared to patients with no increase in Scr (Supplementary Table 8).

#### 3.2.3. Relationship between Scr at Baseline and In-Hospital Adverse Events

Patients with abnormal Scr at baseline were significantly more likely to experience MACE (18.9% vs. 8.6%, *P* < 0.001), in-hospital death (3.2% vs. 0.6%, *P* < 0.001), atrial fibrillation, heart failure, cardiogenic shock, cardiac arrest, stroke, or massive hemorrhage (all *P* < 0.001) compared to patients with normal Scr at baseline (Supplementary Table 9).

## 4. Discussion

This study described the incidence of small increases in Scr and its association with in-hospital outcomes in a large population of patients with STEMI undergoing primary PCI. Findings showed patients with a small increase in Scr (>0.1 to <0.3 mg/dl) within 48 h of hospital admission were significantly more likely to experience MACE or massive hemorrhage (1.23 and 1.45 times higher incidence, respectively) compared to patients with no increase in Scr. Among the 17,503 patients with normal Scr at hospital admission (90.1% of the total study population), the incidence of MACE and massive hemorrhage were 1.34 and 1.53 higher, respectively, in patients with a small increase in Scr within 48 h of hospital admission compared to patients with no increase in Scr. Patients with abnormal Scr at baseline and a small increase in Scr within 48 h of hospital admission were significantly more likely to experience MACE, in-hospital death, atrial fibrillation, heart failure, cardiogenic shock, cardiac arrest, stroke, or massive hemorrhage compared to patients with normal Scr at baseline and a small increase in Scr within 48 h of hospital admission. These data suggest that clinicians should be aware that a small increase in Scr during hospitalization is an important risk factor for poor outcomes in patients with STEMI who undergo primary PCI.

The relationship between small increases in Scr and adverse outcomes is gaining importance in patients with coronary heart disease after cardiac surgery or AMI. The present study used data from 19,424 patients with STEMI undergoing primary PCI from the CCC-ACS Project, which provides real-world evidence pertaining to ACS and interventional therapy in China [14]. A small increase in Scr during the first 48 h of hospital admission was associated with older age, a higher prevalence of hypertension, a faster heart rate, and a lower LVEF. Interestingly, although patients with small increases in Scr during the first 48 h of hospitalization were more likely to use ACEI/ARB and beta blockers, these medications were not risk factors for changes in Scr.

AKI is a serious complication among patients with AMI undergoing primary PCI and is associated with adverse short- and long-term outcomes [1–9]. Increasingly, attention is being paid to patients with small increases in Scr that do not meet the criteria for AKI. In one study, 30-day mortality was highest in patients who had a minimal increase in Scr (0.0–0.5 mg/dl) within 48 hrs after cardiac and thoracic aortic surgery [11]. In a second study conducted in different centers, the same research group substantiated these initial results and showed their findings were not influenced by patients' baseline characteristics, including age, sex, EuroSCORE, LVEF <30%, history of heart failure, diabetes mellitus, history of chronic obstructive pulmonary disease, or operative technique [12]. Notably, these studies included some patients with AKI, defined as an increase in Scr of ≥0.3 mg/dl within 48 h of hospital admission (according to the KDIGO criteria). Consistent with the present study, a previous report showed that patients with STEMI treated with PCI who had a small increase in Scr (0.1–0.3 mg/dl) within 72 h of hospital admission had a twofold increase in the rate of the composite end point of in-hospital adverse events (occurrence of heart failure, atrial fibrillation, need for mechanical ventilation, and in-hospital mortality) (20.3% vs. 9.7%; *P* < 0.001). A small increase in Scr was an independent risk factor for in-hospital adverse outcomes (OR, 1.92; 95% CI, 1.23–2.97; *P* < 0.05) [13].

Scr may increase due to hemodynamic changes leading to reduced renal blood flow and renal dysfunction. The kidney is susceptible to changes in perfusion pressure. When hemodynamic function is compromised, renal blood flow and GFR significantly decrease, resulting in a decline in renal perfusion, a decrease in the creatinine clearance rate, and an increase in Scr. In the present study, we did not evaluate the eGFR because this index is influenced by age and sex and is used to describe different stages of CKD [17, 18]. Patients with a small increase in Scr within 48 h of hospital admission had lower blood pressure and a faster heart rate at admission, compared to patients with no increase in Scr, indicating compromised hemodynamics. Some evidence suggests changes in Scr may be related to the microcirculation [11].

### 4.1. Limitations

This study had several limitations. First, it was a retrospective study with no follow-up; only in-hospital adverse events were observed. As the CCC-ACS Project is a nationwide registry and quality improvement study established among 150 hospitals that focus on the quality of care for patients with ACS, this large real work data set is representative of clinical settings in China. Second, the change in Scr may lag beyond 48 h following hospital admission, which may have led to an underestimation of the true number of patients with STEMI undergoing PCI who experienced a small increase in Scr. Our study will raise awareness that a small increase in Scr during hospitalization in patients with STEMI undergoing primary PCI that does not meet the criteria for AKI is a risk factor for in-hospital adverse events and that this effect was maintained in patients with normal Scr at admission. Third, cause and effect could not be established. Of note, the administration of contrast media has been associated with changes in Scr. In this study, we excluded contrast medium- and drug-induced changes in Scr, suggesting that the small elevations in Scr were related to the development of disease [19, 20].

## 5. Conclusion

A small increase in Scr during hospitalization in patients with STEMI undergoing primary PCI that does not meet the criteria for AKI is a risk factor for in-hospital adverse events. This effect was maintained in patients with normal Scr at admission.

## Figures and Tables

**Table 1:** Primary analysis: risk factors for a small increase in Scr within 48 h of hospital admission in patients with STEMI who underwent primary PCI.

	OR	95% CI	*P* value
Age > 65	1.171	1.119–1.225	<0.001
ACEI/ARB	1.056	0.983–1.135	0.136
Beta-blocker	1.057	0.984–1.135	0.131
Hypertension	1.058	0.982–1.140	0.138
EF < 40%	1.341	1.195–1.504	<0.001
HR	1.005	1.003–1.007	<0.001

Variables included sex, age > 65, hypertension, use of glycoprotein IIb/IIIa inhibitors, use of ACEI/ARB, use of beta-blockers, EF < 40%, SBP, and HR. OR: odds ratios; CI: confidence interval.

**Table 2:** Primary analysis: in-hospital outcomes for patients with STEMI who underwent primary PCI (*n* = 19,424).

	Small increase in Scr within 48 h of hospital admission		*P* value
	Yes (*n* = 5221)	No (*n* = 14203)	
MACE	584 (11.2)	1291 (9.1)	<0.001
Death	41 (0.8)	134 (0.9)	0.301
Stent thrombosis	22 (0.4)	27 (0.2)	0.004
History of AF	174 (3.3)	416 (2.9)	0.146
History of HF	468 (9.0)	918 (6.5)	<0.001
Cardiogenic shock	140 (2.7)	393 (2.8)	0.746
Cardiac arrest	79 (1.5)	181 (1.3)	0.199
Stroke	86 (0.5)	58 (0.4)	0.399
Major bleeding	167 (3.2)	307 (2.2)	<0.001

Data are presented as *n* (%). MACE: major adverse cardiovascular events.

**Table 3:** Primary analysis: risk factors for MACE in patients with STEMI who underwent primary PCI.

	OR	95% CI	*P* value
Small increase in Scr within 48 h of hospital admission	1.168	1.044–1.306	0.006
Female	0.858	0.757–0.974	0.017
Age > 65	1.454	1.357–1.559	<0.001
History of AF	1.591	1.129–2.244	0.008
History of HF	4.573	2.847–7.348	<0.001
IIb/IIIa inhibitors	1.423	1.283–1.579	<0.001
ACEI/ARB	0.887	0.790–0.996	0.043
EF < 40%	2.206	1.902–2.558	<0.001
Killip III-IV	5.770	5.022–6.628	<0.001
SBP	0.988	0.985–0.992	<0.001
HR	1.017	1.014–1.020	<0.001

Variables included small increase in Scr within 48 h of hospital admission, sex, age > 65, hypertension, diabetes, history of AF, history of heart failure, use of glycoprotein IIb/IIIa inhibitors, use of ACEI/ARB, use of beta-blockers, EF < 40%, Killip III-IV, SBP, HR, and previous MI.

**Table 4:** Primary analysis: risk factors for massive hemorrhage in patients with STEMI who underwent primary PCI.

	OR	95% CI	*P* value
Small increase in Scr within 48 h of hospital admission	1.413	1.164–1.715	<0.001
Killip III-IV	2.814	2.184–3.626	<0.001
Age > 65	1.238	1.099–1.396	<0.001
HR	1.013	1.008–1.018	<0.001
IIb/IIIa inhibitors	1.534	1.271–1.852	<0.001
Aspirin	0.491	0.326–0.742	0.001

Variables included small increase in Scr within 48 h of hospital admission, sex, age > 65, hypertension, diabetes, history of AF, history of heart failure, use of glycoprotein IIb/IIIa inhibitors, use of aspirin, use of clopidogrel/ticagrelor, use of ACEI/ARB, use of beta-blockers, EF < 40%, Killip III-IV, SBP, and HR.

## Data Availability

The datasets generated and analyzed during the current study are available from the corresponding author on reasonable request.

## Supplementary Materials

Supplementary Materials Supplementary Table 1: primary analysis: baseline demographic and clinical characteristics of all patients with STEMI who underwent primary PCI (*n* = 19,424). Supplementary Table 2: subgroup analysis: baseline demographic and clinical characteristics of patients with STEMI who underwent primary PCI with normal Scr at hospital admission (*n* = 17,503). Supplementary Table 3: subgroup analysis: risk factors for a small increase in Scr within 48 h of hospital admission in patients with STEMI who underwent primary PCI with normal Scr at hospital admission. Supplementary Table 4: subgroup analysis: in-hospital outcomes in patients with STEMI who underwent primary PCI with normal Scr at hospital admission. Supplementary Table 5: subgroup analysis: risk factors for MACE in patients with STEMI who underwent primary PCI with normal Scr at hospital admission. Supplementary Table 6: subgroup analysis: risk factors for massive hemorrhage in patients with STEMI who underwent primary PCI with normal Scr at hospital admission. Supplementary Table 7: subgroup analysis: baseline demographic and clinical characteristics of patients with STEMI who underwent primary PCI with abnormal Scr at hospital admission (*n* = 1921). Supplementary Table 8: subgroup analysis: in-hospital outcomes in patients with STEMI who underwent primary PCI with abnormal Scr at hospital admission. Supplementary Table 9: subgroup analysis: in-hospital outcomes in patients with STEMI who underwent primary PCI with a small increase in Scr within 48 h of hospital admission, stratified according to Scr at hospital admission (*n* = 19,424).
