# Supplementary material for: A Small Increase in Serum Creatinine within 48 h of Hospital Admission Is an Independent Predictor of In-Hospital Adverse Outcomes in Patients with ST-Segment Elevation Myocardial Infarction Undergoing Primary Percutaneous Coronary Intervention: Findings from the Improving Care for Cardiovascular Disease in the China Project
Source: Cardiol Res Pract. 2023 Mar 28;2023:1374206. doi: 10.1155/2023/1374206 (PMC10072956; doi:10.1155/2023/1374206)
Supplement: Supplementary Materials — Supplementary Table 1: primary analysis: baseline demographic and clinical characteristics of all patients with STEMI who underwent primary PCI (n = 19,424). Supplementary Table 2: subgroup analysis: baseline demographic and clinical characteristics of patients with STEMI who underwent primary PCI with normal Scr at hospital admission (n = 17,503). Supplementary Table 3: subgroup analysis: risk factors for a small increase in Scr within 48 h of hospital admission in patients with STEMI who underwent primary PCI with normal Scr at hospital admission. Supplementary Table 4: subgroup analysis: in-hospital outcomes in patients with STEMI who underwent primary PCI with normal Scr at hospital admission. Supplementary Table 5: subgroup analysis: risk factors for MACE in patients with STEMI who underwent primary PCI with normal Scr at hospital admission. Supplementary Table 6: subgroup analysis: risk factors for massive hemorrhage in patients with STEMI who underwent primary PCI with normal Scr at hospital admission. Supplementary Table 7: subgroup analysis: baseline demographic and clinical characteristics of patients with STEMI who underwent primary PCI with abnormal Scr at hospital admission (n = 1921). Supplementary Table 8: subgroup analysis: in-hospital outcomes in patients with STEMI who underwent primary PCI with abnormal Scr at hospital admission. Supplementary Table 9: subgroup analysis: in-hospital outcomes in patients with STEMI who underwent primary PCI with a small increase in Scr within 48 h of hospital admission, stratified according to Scr at hospital admission (n = 19,424). [file 1374206.f1.doc]

**Supplementary Table 1** Primary analysis: Baseline demographic and clinical characteristics of all patients with STEMI who underwent primary PCI (n = 19,424)

|  | Small increase in Scr within 48 h of hospital admission | | P Value |
| --- | --- | --- | --- |
|  | Yes (n=5221) | No (n=14203) |  |
| **Demographics** | | | |
| Age, y | 61±12 | 60±12 | <0.001 |
| Female (%) | 1005 (19.2) | 2686 (18.9) | 0.595 |
| **Risk factors** | | | |
| Smoking (%) | 2606 (49.9) | 7313 (51.5) | 0.052 |
| **Comorbidities** | | | |
| Previous MI (%) | 233 (4.5) | 666 (4.7) | 0.506 |
| Previous PCI (%) | 227 (4.3) | 686 (4.8) | 0.159 |
| Previous CABG (%) | 12 (0.2) | 25 (0.2) | 0.446 |
| Hypertension (%) | 3194 (61.2) | 8135 (57.3) | <0.001 |
| Dyslipidemia (%) | 4493 (86.1) | 11616 (81.8) | <0.001 |
| Diabetes (%) | 1083 (20.7) | 2978 (21.0) | 0.733 |
| History of AF (%) | 73 (1.4) | 168 (1.2) | 0.229 |
| History of HF (%) | 31 (0.6) | 64 (0.5) | 0.205 |
| **Clinical chemistry** | | | |
| CK-MB First/(ng/ml) | 48.35 (17.00,143.00) | 31.30 (13.00, 102.35) | <0.001 |
| BNP/(pg/ml) | 105.0 (35.00, 323.00) | 108.4 (33.00, 317.00) | 0.803 |
| Hemoglobin /(g/L) | 140±19 | 139±19 | 0.222 |
| TNI/(ng/ml) | 3.99 (0.33,25.04) | 2.27 (0.17, 17.60) | <0.001 |
| Serum creatinine/(umol/L) | 70.30 (59.70,84.00) | 77.00 (65.00,92.00) | <0.001 |
| Echo EF<40% (%) | 495 (9.5) | 989 (7.0) | <0.001 |
| **Clinical characteristics** | | | |
| SBP/mmHg | 128±22 | 126±23 | <0.001 |
| DBP/mmHg | 78±15 | 77±15 | <0.001 |
| HR/bpm | 79±16 | 77±16 | <0.001 |
| Killip III-IV (%) | 331 (6.3) | 897 (6.3) | 0.951 |
| **In-hospital medication** | | | |
| Aspirin (%) | 5095 (97.6) | 13845 (97.5) | 0.671 |
| Clopidogrel/ Ticagrelor (%) | 5141 (98.5) | 13964 (98.3) | 0.464 |
| Beta-blocker (%) | 2955 (56.6) | 7636 (53.8) | <0.001 |
| ACEI/ARB (%) | 2652 (50.8) | 6802 (47.9) | <0.001 |
| Statin (%) | 4988 (95.5) | 13546(95.4) | 0.630 |
| Glycoprotein IIb/IIIa inhibitors (%) | 2706 (51.8) | 6712 (47.3) | <0.001 |

Data are presented as mean ± standard deviation, n (%), or median (interquartile range).

Hypertension was defined as having a history of hypertension, receiving antihypertensive therapy, or having a systolic blood pressure≥140mmHg or diastolic blood pressure ≥90mmHg at hospital admission.

Diabetes mellitus was defined as having a previous or new diagnosis of diabetes mellitus, receiving oral hypoglycemic drug therapy or insulin therapy, or having a HBA1C≥6.5%.

Dislipidemia was defined as having a history of hyperlipidemia, receiving lipid-lowering drugs, or having a serum LDL-C≥1.8mmol/L at hospital admission.

Current smoking was defined as smoking in the preceding 1 year according to the patient’s medical records.

MI: myocardial infarction; PCI: percutaneous coronary intervention; CABG: coronary artery bypass grafting; AF: atrial fibrillation; HF: heart failure; EF: [ejection](javascript:;) [fraction](javascript:;); SBP: systolic blood pressure; DBP: [diastolic](javascript:;) [blood](javascript:;) [pressure](javascript:;); HR: heart rate; ACEI: angiotensin-converting enzyme inhibitor; ARB: angiotensin receptor blocker

**Supplementary Table 2 Subgroup analysis: Baseline demographic and clinical characteristics of patients with STEMI who underwent primary PCI with normal Scr at hospital admission (n = 17,503)**

|  | Small increase in Scr within 48 h of hospital admission | | P Value |
| --- | --- | --- | --- |
|  | Yes (n=4839) | No (n=12664) |  |
| **Demographics** | | | |
| Age, y | 61±12 | 60±12 | <0.001 |
| Female (%) | 948 (19.6) | 2418 (19.1) | 0.455 |
| **Risk factors** | | | |
| Smoking (%) | 2430 (50.2) | 6629 (52.3) | 0.012 |
| **Comorbidities** | | | |
| Previous MI (%) | 209 (4.3) | 584 (4.6) | 0.405 |
| Previous PCI (%) | 208 (4.3) | 597 (4.7) | 0.240 |
| Previous CABG (%) | 10 (0.2) | 23 (0.2) | 0.733 |
| Hypertension (%) | 2908 (60.1) | 7156 (56.5) | <0.001 |
| Dyslipidemia (%) | 4172 (86.2) | 10435 (82.4) | <0.001 |
| Diabetes (%) | 975 (20.1) | 2602 (20.5) | 0.560 |
| History of AF (%) | 63 (1.3) | 142 (1.1) | 0.320 |
| History of HF (%) | 21 (0.4) | 44 (0.3) | 0.400 |
| **Clinical chemistry** | | | |
| CK-MB First/(ng/ml) | 48.90 (17.00,143.63) | 31.00 (13.00, 102.00) | <0.001 |
| BNP/(pg/ml) | 100.00(34.00,300.00) | 100.00(31.00,283.00) | 0.357 |
| Hemoglobin /(g/L) | 140±19 | 140±19 | 0.384 |
| TNI/(ng/ml) | 3.92 (0.33,25.14) | 2.14 (0.16, 17.11) | <0.001 |
| Serum creatinine/(umol/L) | 69.00 (59.00,80.40) | 75.00 (64.00,87.00) | <0.001 |
| Echo LVEF<40% (%) | 443 (9.2) | 806 (6.4) | <0.001 |
| **Clinical characteristics** | | | |
| SBP/mmHg | 128±22 | 127±23 | <0.001 |
| DBP/mmHg | 78±14 | 77±14 | 0.001 |
| HR/bpm | 79±15 | 77±16 | <0.001 |
| Killip III-IV (%) | 265 (5.5) | 647 (5.1) | 0.328 |
| **In-hospital medication** | | | |
| Aspirin (%) | 4722 (97.6) | 12383 (97.8) | 0.430 |
| Clopidogrel/Ticagrelor (%) | 4768 (98.5) | 12471 (98.5) | 0.785 |
| Beta-blocker (%) | 2772 (57.3) | 6961 (55.0) | 0.006 |
| ACEI/ARB (%) | 2466 (51.0) | 6128 (48.4) | 0.002 |
| Statin (%) | 4616 (95.4) | 12112(95.6) | 0.473 |
| Glycoprotein IIb/IIIa inhibitors (%) | 2526 (52.2) | 6051 (47.8) | <0.001 |

Data are presented as mean ± standard deviation, n (%), or median (interquartile range)

Hypertension was defined as having a history of hypertension, receiving antihypertensive therapy, or having a systolic blood pressure≥140mmHg or diastolic blood pressure ≥90mmHg at hospital admission.

Diabetes mellitus was defined as having a previous or new diagnosis of diabetes mellitus, receiving oral hypoglycemic drug therapy or insulin therapy, or having a HBA1C≥6.5%.

Dislipidemia was defined as having a history of hyperlipidemia, receiving lipid-lowering drugs, or having a serum LDL-C≥1.8mmol/L at hospital admission.

Current smoking was defined as smoking in the preceding 1 year according to the patient’s medical records.

MI: myocardial infarction; PCI: percutaneous coronary intervention; CABG: coronary artery bypass grafting; AF: atrial fibrillation; HF: heart failure; EF: [ejection](javascript:;) [fraction](javascript:;); SBP: systolic blood pressure; DBP: [diastolic](javascript:;) [blood](javascript:;) [pressure](javascript:;); HR: heart rate; ACEI: angiotensin-converting enzyme inhibitor; ARB: angiotensin receptor blocker

**Supplementary Table 3** Subgroup analysis: Risk factors for a small increase in Scr within 48 h of hospital admission in patients with STEMI who underwent primary PCI with normal Scr at hospital admission

|  | OR | 95%CI | P Value |
| --- | --- | --- | --- |
| Age>65 | 1.192 | 1.136-1.251 | <0.001 |
| IIb/IIIa inhibitors | 1.222 | 1.142-1.307 | <0.001 |
| Anticoagulation | 1.226 | 1.1214-1.341 | <0.001 |
| ACEI/ARB | 1.064 | 0.988-1.147 | 0.102 |
| Beta-blocker | 1.031 | 0.957-1.111 | 0.420 |
| EF<40% | 1.406 | 1.243-1.590 | <0.001 |
| HR | 1.006 | 1.004-1.008 | <0.001 |

**Supplementary Table 4** Subgroup analysis: In-hospital outcomes in patients with STEMI who underwent primary PCI with normal Scr at hospital admission

|  | Small increase in Scr within 48 h of hospital admission | | P Value |
| --- | --- | --- | --- |
|  | Yes (n=4839) | No (n=12664) |  |
| MACE | 512 (10.6) | 1000 (7.9) | <0.001 |
| Death | 32 (0.7) | 81 (0.6) | 0.873 |
| Stent thrombosis | 21 (0.4) | 22 (0.2) | 0.002 |
| History of AF | 161 (3.3) | 332 (2.6) | 0.012 |
| History of HF | 407 (8.4) | 722 (5.7) | <0.001 |
| Cardiogenic shock | 112 (2.3) | 269 (2.1) | 0.440 |
| Cardiac Arrest | 62 (1.3) | 131 (1.0) | 0.162 |
| Stroke | 23 (0.5) | 40 (0.3) | 0.115 |
| Major Bleeding | 140 (2.9) | 235 (1.9) | <0.001 |

Data are presented as n (%)

**Supplementary Table 5** Subgroup analysis: Risk factors for MACE in patients with STEMI who underwent primary PCI with normal Scr at hospital admission

|  | OR | 95%CI | P Value |
| --- | --- | --- | --- |
| Small increase in Scr within 48 h of hospital admission | 1.247 | 1.107-1.406 | <0.001 |
| Female | 0.824 | 0.718-0.944 | 0.005 |
| Age>65 | 1.428 | 1.321-1.544 | <0.001 |
| History of AF | 1.626 | 1.110-2.382 | 0.013 |
| History of HF | 5.376 | 3.030-9.539 | <0.001 |
| Diabetes | 1.154 | 1.009-1.319 | 0.031 |
| IIb/IIIa inhibitors | 1.437 | 1.283-1.611 | <0.001 |
| ACEI/ARB | 0.871 | 0.768-0.988 | 0.031 |
| Beta-blocker | 0.982 | 0.867-1.111 | 0.770 |
| EF<40% | 2.230 | 1.892-2.629 | <0.001 |
| Killip III-IV | 5.782 | 4.929-6.782 | <0.001 |
| SBP | 0.990 | 0.986-0.994 | <0.001 |
| HR | 1.018 | 1.014-1.021 | <0.001 |

Variables included: small increase in Scr within 48 h of hospital admission, sex, age>65, history of AF, history of heart failure, use of glycoprotein IIb/IIIa inhibitors, use of aspirin, use of clopidogrel/ ticagrelor, use of ACEI/ARB, use of beta-blockers, EF<40%, Killip III-IV, SBP, HR

**Supplementary Table 6** Subgroup analysis: Risk factors for massive hemorrhage in patients with STEMI who underwent primary PCI with normal Scr at hospital admission

|  | OR | 95%CI | P Value |
| --- | --- | --- | --- |
| Small increase in Scr within 48 h of hospital admission | 1.445 | 1.166-1.791 | 0.001 |
| Killip III-IV | 2.685 | 1.976-3.648 | <0.001 |
| Age>65 | 1.265 | 1.103-450 | 0.001 |
| HR | 1.013 | 1.007-1.019 | <0.001 |
| IIb/IIIa inhibitors | 1.660 | 1.343-2.053 | <0.001 |
| Aspirin | 0.505 | 0.305-0.837 | 0.008 |

Variables included: small increase in Scr within 48 h of hospital admission, sex, age>65, use of glycoprotein IIb/IIIa inhibitors, use of aspirin, use of clopidogrel/ ticagrelor, EF<40%, Killip III-IV, HR

**Supplementary Table 7 Subgroup analysis: Baseline demographic and clinical characteristics of patients with STEMI who underwent primary PCI with abnormal Scr at hospital admission (n=1921)**

|  | Small increase in Scr within 48 h of hospital admission | | P Value |
| --- | --- | --- | --- |
|  | Yes (n=382) | No (n=1539) |  |
| **Demographics** | | | |
| Age, y | 66±13 | 65±13 | <0.001 |
| Female (%) | 57 (14.9) | 268 (17.4) | 0.245 |
| **Risk factors** | | | |
| Smoking (%) | 176 (46.1) | 684 (44.4) | 0.567 |
| **Comorbiditoes** | | | |
| Previous MI (%) | 24 (6.3) | 82 (5.3) | 0.465 |
| Previous PCI (%) | 19 (5.0) | 89 (5.8) | 0.539 |
| Previous CABG (%) | 2 (0.5) | 2 (0.1) | 0.179 |
| Hypertension (%) | 286 (74.9) | 979 (63.6) | <0.001 |
| Dislipdemia (%) | 321 (84.0) | 1181 (76.7) | 0.002 |
| Diabetes (%) | 108 (28.3) | 376 (24.4) | 0.122 |
| History of AF (%) | 10 (2.6) | 26 (1.7) | 0.231 |
| History of HF (%) | 10 (2.6) | 20 (1.3) | 0.063 |
| **Clinical chemistry** | | | |
| CK-MB First/(ng/ml) | 44.50 (15.78,137.61) | 33.70 (13.00, 108.68) | 0.054 |
| BNP/(pg/ml) | 314.00 (87.00,761.00) | 261.00 (65.45,870.71) | 0.546 |
| Hemoglobin /(g/L) | 132±21 | 134±23 | 0.304 |
| TNI/(ng/ml) | 4.06 (0.37,25.00) | 4.03 (0.27,27.09) | 0.645 |
| Serum creatinine/(umol/L) | 124.00 (105.28,144.80) | 126.00 (110.00,155.00) | 0.003 |
| Echo LVEF<40% (%) | 52 (13.6) | 183 (11.9) | 0.358 |
| **Clinical characteristics** | | | |
| SBP/mmHg | 130±25 | 122±27 | <0.001 |
| DBP/mmHg | 78±16 | 77±17 | <0.001 |
| HR/bpm | 79±19 | 77±20 | 0.237 |
| Killip III-IV (%) | 66 (17.3) | 250 (16.2) | 0.626 |
| **In-hospital medication** | | | |
| Aspirin (%) | 373 (97.6) | 1462 (95.0) | 0.025 |
| Clopidogrel/ Ticagrelor (%) | 373 (97.6) | 1493 (97.0) | 0.507 |
| Beta-blocker (%) | 183 (47.9) | 675 (43.9) | 0.155 |
| ACEI/ARB (%) | 186 (48.7) | 674 (43.8) | 0.085 |
| Statin (%) | 372 (97.4) | 1434 (93.2) | 0.002 |
| Glycoprotein IIb/IIIa inhibitors (%) | 180 (47.1) | 661 (42.9) | .0141 |

Data are presented as mean ± standard deviation, n (%), or median (interquartile range)

Hypertension was defined as having a history of hypertension, receiving antihypertensive therapy, or having a systolic blood pressure≥140mmHg or diastolic blood pressure ≥90mmHg at hospital admission.

Diabetes mellitus was defined as having a previous or new diagnosis of diabetes mellitus, receiving oral hypoglycemic drug therapy or insulin therapy, or having a HBA1C≥6.5%.

Dislipidemia was defined as having a history of hyperlipidemia, receiving lipid-lowering drugs, or having a serum LDL-C≥1.8mmol/L at hospital admission.

Current smoking was defined as smoking in the preceding 1 year according to the patient’s medical records.

MI: myocardial infarction; PCI: percutaneous coronary intervention; CABG: coronary artery bypass grafting; AF: atrial fibrillation; HF: heart failure; EF: [ejection](javascript:;) [fraction](javascript:;); SBP: systolic blood pressure; DBP: [diastolic](javascript:;) [blood](javascript:;) [pressure](javascript:;); HR: heart rate; ACEI: angiotensin-converting enzyme inhibitor; ARB: angiotensin receptor blocker

**Supplementary Table 8** Subgroup analysis: In-hospital outcomes in patients with STEMI who underwent primary PCI with abnormal Scr at hospital admission

|  | Small increase in Scr within 48 h of hospital admission | | P Value |
| --- | --- | --- | --- |
|  | Yes (n=382) | No (n=1539) |  |
| MACE | 72 (18.8) | 291 (18.9) | 0.979 |
| Death | 9 (2.4) | 53 (3.4) | 0.282 |
| Stent thrombosis | 1 (0.3) | 5 (0.3) | 1.000 |
| History of AF | 13 (3.4) | 84 (5.5) | 0.101 |
| History of HF | 61 (16.0) | 196 (12.7) | 0.097 |
| Cardiogenic shock | 28 (7.3) | 124 (8.1) | 0.637 |
| Cardiac Arrest | 17 (4.5) | 50 (3.2) | 0.252 |
| Stroke | 3 (0.8) | 18 (1.2) | 0.783 |
| Major Bleeding | 27 (7.1) | 72 (4.7) | 0.059 |

Data are presented as n (%)

**Supplementary Table 9** Subgroup analysis: In-hospital outcomes in patients with STEMI who underwent primary PCI with a small increase in Scr within 48 h of hospital admission, stratified according to Scr at hospital admission (n = 19,424)

|  | Serum creatinine at hospital admission | | P Value |
| --- | --- | --- | --- |
|  | Abnormal (n=1921) | Normal (n=17503) |  |
| MACE | 363 (18.9) | 1512 (8.6) | <0.001 |
| Death | 62 (3.2) | 113 (0.6) | <0.001 |
| Stent thrombosis | 6 (0.3) | 43 (0.2) | 0.580 |
| History of AF | 97 (5.0) | 493 (2.8) | <0.001 |
| History of HF | 257 (13.4) | 1129 (6.5) | <0.001 |
| Cardiogenic shock | 152 (7.9) | 381 (2.2) | <0.001 |
| Cardiac Arrest | 67 (3.5) | 193 (1.1) | <0.001 |
| Stroke | 21 (1.1) | 63 (0.4) | <0.001 |
| Major Bleeding | 99 (5.2) | 375 (2.1) | <0.001 |

Data are presented as n (%)
